# Cobalt–Nitrogen Co-Doped Carbon as Highly Efficient Oxidase Mimics for Colorimetric Assay of Nitrite

**DOI:** 10.3390/bios13070748

**Published:** 2023-07-20

**Authors:** Dalei Lin, Shuzhi Wu, Shushu Chu, Yizhong Lu

**Affiliations:** 1 School of Materials Science and Engineering, University of Jinan, Jinan 250022, China; 2 Shandong Academy of Preventive Medicine, Shandong Center for Disease Control and Prevention, Jinan 250014, China

**Keywords:** cobalt–nitrogen co-doped carbon (Co-N-C), oxidase mimic, nitrite determination, nanozyme

## Abstract

Transition metal-N-doped carbon has been demonstrated to mimic natural enzyme activity; in this study, cobalt–nitrogen co-doped carbon (Co-N-C) nanomaterial was developed, and it could be an oxidase mimic. Firstly, Co-N-C with oxidase-like activity boosts the chromogenic reaction of 3,3′,5,5′-tetramethylbenzidine (TMB) to produce the oxidized TMB (oxTMB). And the aromatic primary amino group of oxTMB reacts with nitrite (NO_2_^−^) to form diazo groups. Based on this background, we developed a cascade system of a Co-N-C-catalyzed oxidation reaction and a diazotization reaction for nitrite determination. The low detection limit (0.039 μM) indicates that Co-N-C is superior compared with the vast majority of previously reported nitrite assays. This study not only provides a novel nanozyme with sufficiently dispersed active sites, but it also further applies it to the determination of nitrite, which is expected to expand the application of nanozymes in colorimetric analysis.

## 1. Introduction

Enzymes are biocatalysts with high catalytic activity and specificity, whereas high price and easy inactivation limit their application and development [1,2,3,4]. Therefore, artificial enzymes were first reported by Yan et al. in 2007 [5], revealing the intrinsic biological effects and new properties of nanomaterials, enriching the investigation of artificial enzymes and expanding the application range of nanomaterials. Nanozymes are nanomaterials with enzymatic characteristics, and they have many benefits such as low cost, high activity, and mass preparation, which have caused a lot of attention [6]. Recently, different from traditional nanomaterials, transition metal-nitrogen-doped carbon (M-N-C) nanocatalysts have been evaluated as catalysts with great prospects in many fields because they have sufficiently exposed active sites and thus excellent activity, coupled with particularly low preparation costs. The M-N-C catalyst maximizes the utilization rate of metal atoms in terms of size, structure, and composition, and improves the catalytic performance by regulating the interaction between metal atoms and the support. In other words, optimizing the electronic structure to regulate the adsorption and desorption of substrates and intermediates to boost the improvement of enzyme-like activity [7,8,9]. So far, although many reports of mimetic enzyme materials focusing on Co, low atomic utilization and ambiguous active sites remain obstacles that the majority of nanozymes struggle to overcome in catalytic reactions [10,11,12,13]. Therefore, the enhancement of the loading and exposure of the active sites plays a crucial role in achieving high activity catalyzed by nanozymes. Consequently, it is of great significance to prepare Co-N-C catalysts with highly dispersed Co-N_x_ active sites and study their enzymatic activities.

As we all know, nanozymes with excellent activity and stability have been used in many fields related to human survival and life, such as food safety, environmental monitoring, clinical medicine, etc. In food processing, nitrite is commonly employed as a preservative or colorant, thereby extending the food expiry time of food and enriching the taste [14]. However, residual nitrite can give rise to a high accumulation of hemosiderin, which can be harmful to health [15]. Furthermore, the drinking water guideline for nitrite intake was 3 mg L^−1^ (the molar concentration is approximately 65 μM) has been set by the World Health Organization [16], so the development of an efficient and accurate test method plays an essential role in ensuring food safety. Until now, analytical approaches for detecting nitrite have been developed, such as electrochemical [17], fluorescence [18], colorimetric [19] analysis, and so on. Nevertheless, the single signal involved in these measures is susceptible to environmental interference [16], and it is important to explore new monitoring methods. Taking into account the above requirement, we chose TMB as the substrate in catalytic reaction similarities to natural enzymes [20]; oxTMB is generated based on oxidase-like characteristics involved in Co-N-C, and its aromatic primary amino group reacts with nitrite to form diazo groups to achieve the detection of nitrite in food. Subsequently, oxidation and diazotization reactions form a cascade system. Furthermore, UV-vis spectra with bimodal signals reduce systematic errors caused by the experimental environment.

Herein, the Co-N-C nanocatalyst was satisfactorily prepared, which not only maximizes the availability of metal atoms, but also exposes affluent metal active sites and, therefore, exhibits magnificent oxidase-like activity. Further combined with the diazotization reaction, a bimodal ratiometric colorimetric analysis for accurate detection of nitrite was appropriately explored.

## 2. Experimental Section

### 2.1. Preparation of Co-N-C and N-C

Co-N-C was prepared according to a general ligand-mediated strategy [21]. In detail, (CH_3_COO)_2_Co·4H_2_O (18.8 mg) and 1,10-Phenanthroline (94.99 mg) were added to 2 mL ethanol and stirred. After stirring at room temperature for 20 min, carbon black (69.6 mg) was added into the above solution, followed by constant stirring and heating in an oil bath at 60 °C for 4 h. Subsequently, the resulting dispersion was then placed in an oven at 80 °C to evaporate the ethanol to obtain dry solid powder, then placed in a ceramic boat and heated at 800 °C at a rate of 10 °C min^−1^ under an Ar atmosphere and kept for 2 h. Then, the obtained materials were cooled naturally to yield Co-N-C samples. A control sample was synthesized through the same fabrication process as that for Co-N-C samples but without the step of Co element incorporation (denoted as N-C).

### 2.2. Probing the Diazotization Reaction of oxTMB

To explore the diazotization between nitrite and oxTMB generating from the Co-N-C + TMB system, 20 μL Co-N-C solution, 20 μL TMB substrate, and 940 μL of acetate buffer were mixed thoroughly. Immediately after 5 min of reaction at room temperature, 20 µL nitrite aqueous solution (5 mM) was added to the above mixture, the reaction is continued for 20 min, and the absorbance change of the mixed solution was recorded by UV-vis spectrophotometer.

### 2.3. Ratiometric Colorimetric Analysis of Nitrite

In total, 20 μL TMB substrate and 20 μL Co-N-C solution were mixed thoroughly. After the substrate was oxidized for 5 min, various volumes of nitrite aqueous solution (5 mM) were added immediately to the above mixed solution in a gradient, and the UV-vis spectra were measured immediately. Furthermore, to verify the applicability of the Co-N-C + TMB + NO_2_^−^ system, sausages and salted quail eggs were pretreated and experimented on in light of the China National Standard (GB 5009.33-2016) [16]. Standard nitrite solutions of different concentrations were then added to these real samples for measurement.

## 3. Result and Discussion

### 3.1. Synthesis and Characterization of Co-N-C

**Figure 1:**
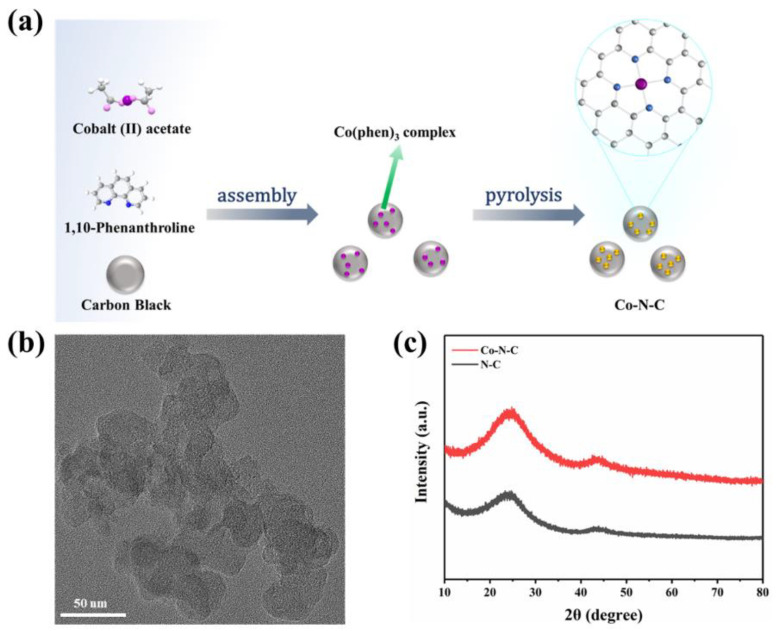
(**a**) A scheme for the synthesize of Co-N-C; (**b**) TEM images of Co-N-C; (**c**) XRD patterns of the Co-N-C and N-C.

Based on a previous report by Zhang et al. [21], according to a general ligand-mediated strategy, we used (CH_3_COO)_2_Co·4H_2_O as a metal precursor to provide cobalt ions, then complexed with 1,10-phenanthroline and loaded on carbon black. The modified carbon was obtained, which was subsequently pyrolyzed in an argon atmosphere at 800 °C, generating the formation of Co-N-C (Figure 1a). According to previously reported articles, after the introduction of nitrogen-doped carbon, Co was coordinated fourfold by N atoms obtaining CoN_4_ on Co-N-C. The cobalt–nitrogen co-doped carbon with the well-defined CoN_4_ coordination structure could promote the direct formation of enzyme-like active sites.

**Figure 2:**
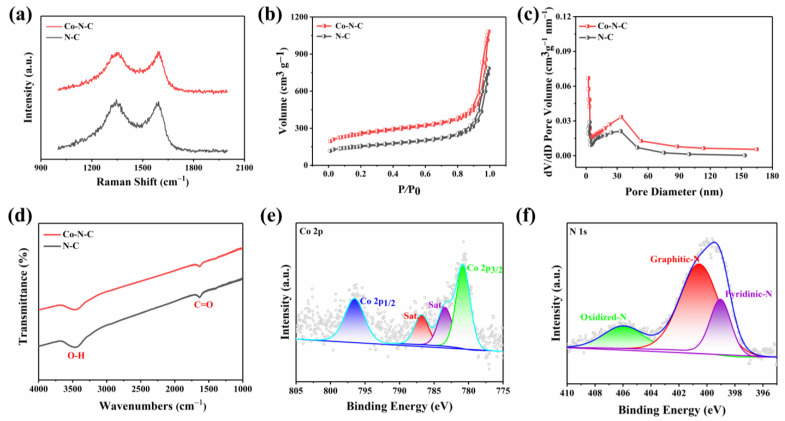
(**a**) Raman spectra of the Co-N-C and N-C; (**b**) N_2_ adsorption/desorption isotherms and (**c**) corresponding pore size distribution of the NC and Co-N-C; (**d**) FTIR spectra of the Co-N-C and N-C; (**e**) Co 2p and (**f**) N 1s spectra of Co-N-C.

The morphology of the prepared Co-N-C was observed by transmission electron microscopy (TEM), and no metal particles and clusters were seen on the carbon black carrier (Figure 1b and Figure S1), indicating that Co was well dispersed. Moreover, the powder X-ray diffraction (XRD) further proved the Co-N-C without metal. The XRD patterns of both Co-N-C and N-C showed only two characteristic peaks at 25.2° and 43.1°, corresponding to the (002) and (101) planes of graphite [21] (Figure 1c). In addition, the introduction of Co and N resulted in lattice distortion defects in carbon, so that the wide diffraction peak of the intact graphite plane deviated from a tiny angle. No significant diffraction peak of the crystalline Co species was present in Co-N-C, validating that Co is incorporated into carbon in an amorphous form, which is incredibly consistent with the TEM image. The Raman spectrum for Co-N-C with two peaks around 1580 cm^−1^ and 1350 cm^−1^, which can be ascribed to graphitic sp^2^ carbon (G band) and disordered sp^3^ carbon (D band) [22], respectively. The *I*_D_/*I*_G_ ratio for Co-N-C (2.50) was higher than that of the original carbon black (1.95) (Figure S2), indicating that the defective carbon content of the carbon carrier increased during the pyrolysis process for prepared Co-N-C. The *I*_D_/*I*_G_ ratio of N-C (2.44) is slightly lower than that of Co-N-C (2.50) (Figure 2a), which means that there may be Co-induced carbon defects in the sample [22]. Such abundant defects are believed to enhance oxidase-like activities. As shown in Figure 2b,c, the specific surface values of Co-N-C and N-C are 557.67 and 550.40 m^2^ g^−1^, and the pore diameters are 1.67 and 1.70 nm, respectively. The larger specific surface area of Co-N-C with mesoporous provides more active sites for enhancing oxidase-like activities. This may be due to an increase in defects due to the introduction of Co atoms, which leads to an increase in the specific surface area of the catalyst [23,24]. The consequence showed that the mimicked enzyme activity of Co-N-C can be greatly improved by exposing more active sites by increasing the specific surface area. The Fourier transform infrared (FTIR) spectrum shows that Co-N-C and N-C are rich in functional groups [25]. Absorption peaks at 3442 cm^−1^, 1653 cm^−1^, are separately ascribed to O-H, C=O. (Figure 2d). Meanwhile, the surface chemical composition and elemental bonding configurations of Co-N-C were analyzed by XPS analysis, and the characteristic peaks of Co, N, C, O were systematically discovered. As can be seen from the spectra (Figure 2e), the Co 2p spectra exhibit two main peaks at 796.5 and 780.8 eV, suggesting the presence of Co species in the Co-N-C sample. The peaks can be attributed to Co^0^ and Co^2+^ states [26]. The N 1s spectra can be deconvoluted into three types, oxidized-N (406.0 eV), graphitic-N (400.6 eV), and pyridinic-N (399.0 eV) [27] (Figure 2f). The formation of pyridinic N was attributed to coordination with cobalt, while the predominance of graphitic N was the graphitization of the precursor 1,10-phenanthroline [28]. The graphitic N would have an impact on the geometric and electronic structures of the carbon carriers meanwhile pyridinic N has a remarkable probability of bonding with single Co atoms, forming CoN_4_. Except for Co and N, the C and O of XPS were also detected on the catalyst surface. The C1s spectrum was deconvoluted into two peaks, originating from C-N species and C-C or C=C (neutral carbon and adventitious hydrocarbons), respectively [29,30] (Figure S3a). The O 1s spectrum could be deconvoluted into two peaks by the binding energy of 531.5 and 532.7 eV (Figure S3b), corresponding to ketonic C=O groups and C-groups [22], respectively. When the sample enters a high vacuum to record the XPS spectra, no Co-O peaks appear in the O 1s as a result of the disappearance of weakly adsorbed oxygen molecules.

### 3.2. Oxidase-like Activities of Co-N-C

**Figure 3:**
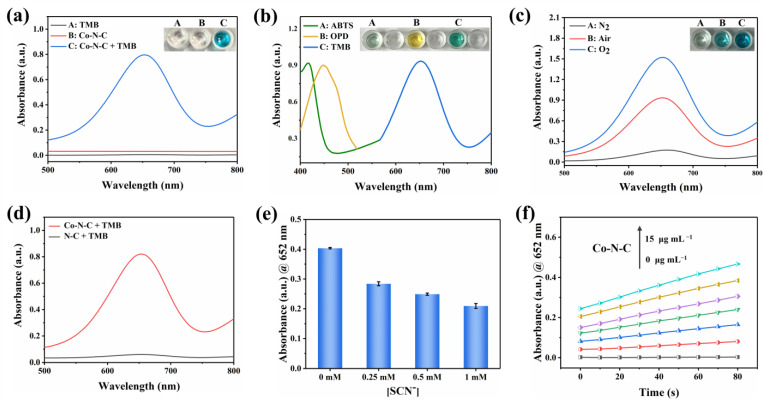
UV-vis spectra of (**a**) various reaction systems, (**b**) Co-N-C catalyzed oxidation of ABTS, OPD, and TMB, (**c**) Co-N-C oxidizing TMB under N_2_, Air and O_2_ saturation conditions, and (**d**) Co-N-C and N-C. (**e**) Changes of UV-vis absorbance intensities of Co-N-C + TMB solution after the addition of different concentrations of SCN^−^. (**f**) Concentration optimization of Co-N-C, (The colored lines represent the concentration of the nanozyme, in order of 0, 2.5, 5.0, 7.5, 10, 12.5, and 15 μg mL^−1^). Error bars represent the standard deviation of three parallel measurements.

Co-N-C has excellent oxidase-like activity due to its advantages of maximizing metal utilization and exposing the active site to the carbon carrier [31]. Inevitably, the oxidase-like activity of Co-N-C was demonstrated with the chromogenic substrate TMB [20]. As can be seen in Figure 3a, when Co-N-C and TMB coexist, Co-N-C oxidizes the colorless TMB to blue oxTMB related to a distinct UV-characteristic absorption peak that appears at 652 nm. In contrast, there is no significant absorbance in the wavelength range of 500–800 nm in the presence of TMB alone, demonstrating the important role of Co-N-C as an oxidase mimic in catalyzing substrate oxidation. As shown in Figure 3b, colorless ABTS and OPD are also oxidized by Co-N-C, producing corresponding green and yellow colors with characteristic absorption peaks at 416 nm and 448 nm, respectively. The oxidase-like activity of Co-N-C was further proven by the oxidation of two other typical chromogenic substrates. It can be seen that the oxidase-like activity of Co-N-C is inhibited in the N_2_-saturated atmosphere, but the activity is significantly enhanced in the O_2_-saturated atmosphere, indicating dissolved oxygen plays an extremely important action in the catalytic oxidation of TMB (Figure 3c). Compared with Co-N-C nanozyme, the N-C nanocatalyst did not exhibit significant oxidase-like activity (Figure 3d). To further study the active site of Co-N-C nanozyme, the effect of thiocyanate ions (SCN^−^) on its oxidase-like activity was investigated owing to the fact that SCN^−^ can poison CoN_x_-centered catalytic sites under acidic conditions [32]. As shown in Figure 3e, the characteristic absorption peak at 652 nm is obviously decreased after the SCN- pretreatment of Co-N-C. This implies that the enzymatic activity of Co-N-C is irreversibly inhibited by SCN^−^, which also shows that the main active site for mimicked oxidase is Co-N_x_. To obtain a suitable absorbance value for evaluating oxidase-like activity, the concentration optimization experiment of Co-N-C catalyst was performed. As shown in Figure 3f, the concentration is too low to prove the activity of the sample well, while the background color of the catalyst with too high concentration is darker, which will affect the absorbance value of the characteristic peaks. Therefore, a Co-N-C catalyst with a concentration of 10 μg mL^−1^ was finally selected for subsequent testing.

**Figure 4:**
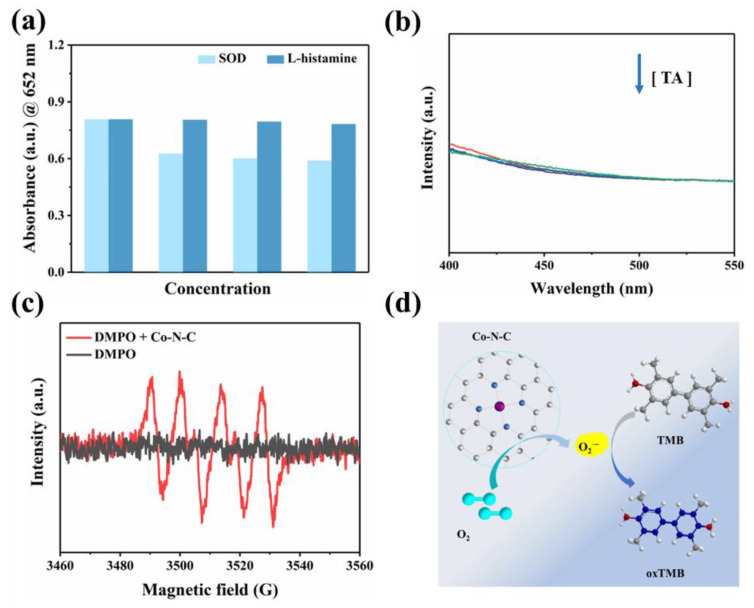
(**a**) UV-vis spectra of the Co-N-C-catalyzed oxidation after addition of ROS scavengers; (**b**) Fluorescence spectra of the Co-N-C + TMB system with gradient concentration of TA, (The colored lines represent the concentration of TA, in order of 0, 2, 4, and 8 mM); (**c**) The EPR spectra of DMPO + Co-N-C methanol solution; (**d**) Schematic diagram of TMB oxidation catalyzed by Co-N-C.

**Figure 5:**
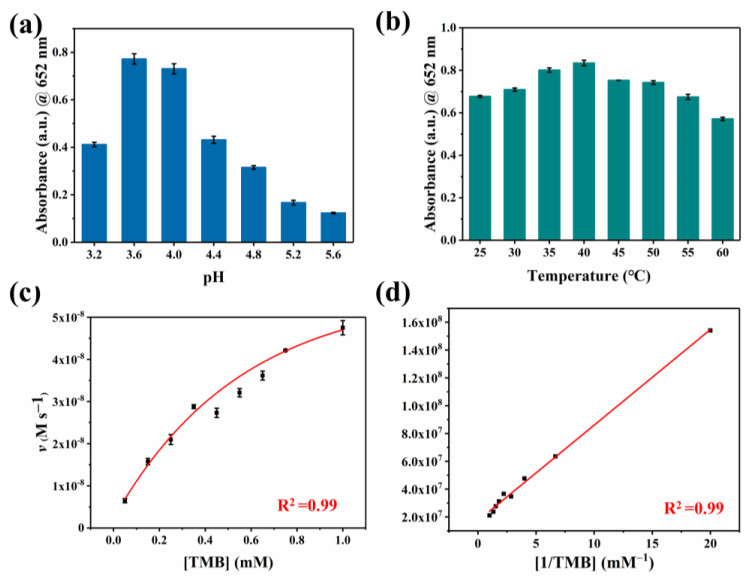
Oxidase-like activities of the Co-N-C dependent on (**a**) pH, (**b**) temperature. (**c**) Steady-state kinetics analysis of Co-N-C and (**d**) corresponding Lineweaver–Burk curve.

To our knowledge, oxygen plays a crucial role as an electron acceptor in the catalytic oxidation of Co-N-C [33,34,35,36]. The activated O_2_ was adsorbed on the surface of the catalyst to generate reactive oxygen species (ROS). In terms of theory, the adsorbed O_2_ may evolve into several ROS in oxidase simulation reactions, including hydroxyl radicals (·OH), superoxide anion (O_2_^·−^), singlet oxygen (^1^O_2_), and hydrogen peroxide (H_2_O_2_) [37,38,39]. Therefore, we performed free radical scavenging exploration and electron paramagnetic resonance (EPR) detection systems to prove the types of ROS generated in the Co-N-C-involved mimicked enzyme reaction to better understand the reaction mechanism. Superoxide dismutase (SOD), the scavenger for O_2_^·−^, can evidently suppress the catalytic reaction (Figure 4a). Furthermore, EPR spetra were performed with 5,5-dimethyl-1-pyrroline N-oxide (DMPO) in methanol solution. As shown in Figure 4c, the characteristic triplet peaks in the EPR plot proved the generation of O_2_^·−^. Subsequently, the scavengers L-histidine and 1,4-dicarboxybenzene (TA) are introduced for ^1^O_2_ and ·OH, respectively. However, these two scavengers did not exert a significant inhibitory effect on oxidase-like activity (Figure 4a,b). Based on the above experimental conclusions, the O_2_^·−^ is the most active species in the reaction system and it accounts for the oxidation of TMB combining with the scavenger and EPR results. (Figure 4d). In fact, the catalytic capability of Co-N-C is pH-, and it is temperature-dependent, resembling natural enzymes. The catalytic activity of Co-N-C elevated initially but decreased next when the buffer is in the range of pH 3.2–pH 5.6, arriving at the optimal pH = 3.6 (Figure 5a). As can be seen in Figure 5b, over a wide temperature range (25 to 55 °C), the optimum activity is at 40 °C, which favors the practicability of the Co-N-C.

Typically, we calculate the steady-state kinetic parameters (i.e., *K*_m_ and *V*_max_) based on kinetic assays to quantitatively assess the oxidase-like activity of Co-N-C. Compared with previously reported Cobalt-based oxidases, the *K*_m_ of Co-N-C is smaller (0.39 mM, Table S1), which shows it is a better affinity that gives rise to superior catalytic efficiency. This excellent oxidase activity is due to atomically dispersed Co-N_4_ active sites and extremely high atomic utilization.

### 3.3. Cascade of Oxidase-like Catalysis and Diazotization

**Figure 6:**
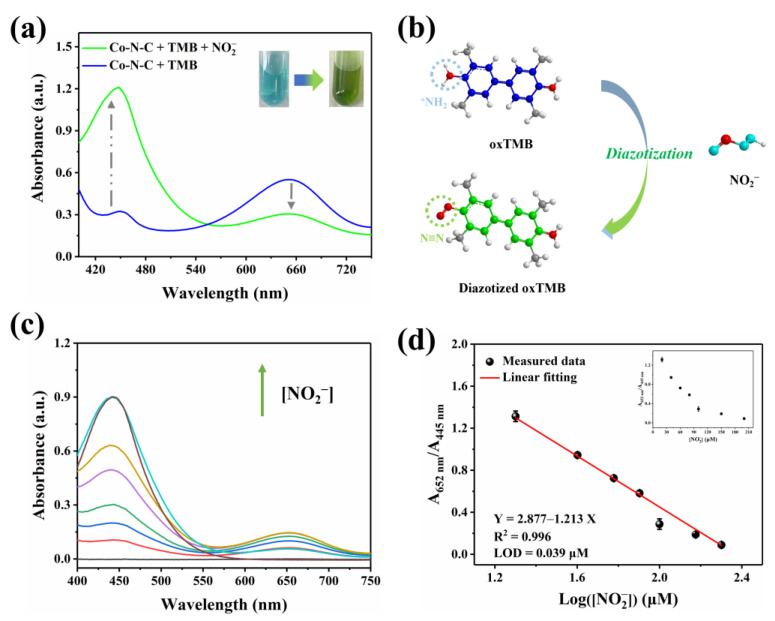
(**a**) Effect of NO_2_^−^ on the UV-vis absorption signal; (**b**) Schematic diagram of diazotization of nitrite with oxTMB; (**c**) UV-vis spectra of Co-N-C + TMB + NO_2_^−^ with NO_2_^−^ at various levels, (The colored lines represent the concentration of NO_2_^−^, in order of 20, 40, 60, 80, 100, 150, and 200 μM); (**d**) Linear relationship between the value of *A*_652_/*A*_445_ and the value of Log([NO_2_^−^]).

Nitrite (NO_2_^−^) is a common nitrogenous compound and widely exists in nature. Superfluous NO_2_^−^ can integrate with hemoglobin, which triggers severe methemoglobin accumulation inside the body [40,41,42,43,44,45]. In this work, after NO_2_^−^ is introduced into the Co-N-C + TMB reaction system, the oxTMB obtained by oxidation would undergo diazotization reaction with it. As shown in Figure 6a, the UV-vis absorption peak at 652 nm decreases, while the signal at 445 nm increases; the color of the solution is converted from blue to green. Therefore, the investigation is known as a cascade process for NO_2_^−^ detection, which will effectively detect the concentration of NO_2_^−^ in a bimodal ratiometric colorimetric mode. By introducing dual characteristic peaks to achieve the output of the ratiometric signal, interference from environmental factors can be reduced, and some systematic errors can be eliminated, thereby improving the reliability of detection. This dual signal occurs due to the aromatic primary amine group of oxTMB reacting with nitrite to form the diazo group (Figure 6b). According to the previous report on L-Ascorbic acid (AA) [46], inhibition of the oxidation of TMB can be introduced to further explore the diazotization interaction between nitrite and oxTMB. Figure S4 shows that the intermediate ester product oxTMB is obtained due to the enzymatic nature of Co-N-C [20]. After AA is subsequently added, oxTMB is entirely reduced [47], and no clearly discernible absorbance was observed in the wavelength range of 400–750 nm. Nevertheless, with the presence of nitrite, oxTMB intermediate reacts with nitrite to form diazotized oxTMB, but AA cannot completely reduce it to the original TMB [16]. As a matter of fact, the hemiquinone-imine structure of diazotized oxTMB can be chemically reduced by AA to the amino structure of aromatic hydrocarbons, while the diazo group cannot be reduced due to its chemical stability. The result manifests its diazotization interaction that occurred between nitrite and oxTMB species.

### 3.4. Co-N-C Applied in the Ratiometric Colorimetric Assay of Nitrite

Nitrite with the capacity to induce opposite changes in two signals in the Co-N-C + TMB system with good specificity, provides a measure for the quantitative determination of nitrite. To achieve optimal detection capabilities, the diazotization time of nitrite and oxTMB is maintained for 20 min, which is on the wavelength of the 652 nm and 445 nm (*A*_652_/*A*_445_) gradually becomes saturated after 20 min (Figure S5). As shown in Figure 6c, with optimal experimental conditions, the consumption of oxTMB leads to the reduction in the absorbance peak at 652 nm following the contribution of nitrite. Correspondingly, the generation of diazotized oxTMB gives rise to the augment of the signal at 445 nm as well. In accordance with the performance, the ratio of *A*_652_/*A*_445_ progressively decreases alongside the elevation in nitrite concentration. A satisfactory linear relationship is further found between *A*_652_/*A*_445_ and the Log([NO_2_^−^]) in the range of 20–200 μM. Accordingly, the linear fitting equation is Y = 4.38 − 1.89X (μM, R^2^ = 0.99) (Figure 6d), and the detection limit (LOD) is 0.039 μM (S/N = 3). In fact, TMB reacts directly with nitrite to quantitatively detect it. As shown in Figure S6, a linear relationship is observed in the concentration range of 20–200 µM, and the LOD is 0.17 µM. Table S2 shows the superiority of ratiometric colorimetry in terms of responsiveness and detection limits compared to previously reported single-signal assays for the detection of nitrite. In particular, the sensitivity and detection limits of this ratiometric detection approach fully meet the demand of human daily life for detecting nitrite in drinking water and food.

Among some considerable indexes affecting nitrite determination, high sensitivity, anti-interference, and high selectivity should be considered. Therefore, various potential interferences were measured in this study, including common ions and biomolecules. Subsequently, the results showed that the influence of these common disturbing species on the bimodal ratiometric detection mode is negligible (Figure S8), which indicates the analytical methods established has significant anti-interference properties.

Additionally, to verify the practicality of the above analysis, it was applied to the detection of nitrite in preserved products such as sausages and salted quail eggs under real environmental conditions. As shown in Table 1, the recovery percent of the ratiometric colorimetric is 100.3–110.8% (RSD ≤ 4.4%), which illustrates the bimodal ratio measurement with superior feasibility in quantifying nitrite content in actual samples, and it is expected to be widely used to analyze nitrite in food needed by human daily life.

## 4. Conclusions

In summary, we successfully prepared Co-N-C by a general ligand-mediated strategy, which maximized metal utilization with low cost in the preparation of nanomaterials. Since CoN_4_ is the active site in Co-N-C, the surface free energy of Co-N-C increases dramatically, coupled with quantum size effects and the influence of unsaturated coordination environment, which both give this mimicked enzyme species high oxidase-like activity, high stability, and selectivity. In this study, TMB was used as a suitable colorimetric substrate, and the mimicked enzyme reaction of Co-N-C was further cascaded with the diazotization reaction, so satisfactory results were achieved in the detection of nitrite. The novel cobalt-based nanozyme with an extremely high metal atom utilization rate has great practical significance, and it further expands the use of nanozymes in bioanalytical sensing.

## Figures and Tables

**Table 1:** Determination of nitrite in samples.

Sample	Added (µM)	Detected (µM)	Recovery (%)	RSD (%, *n* = 3)
	0	7.44	NA	1.3
Pickled quail eggs	50	59.06	110.8	1.7
	100	108.97	108.9	4.4
	0	0.49	NA	0.1
Preserved sausage	50	51.05	102.1	1.5
	100	100.25	100.3	0.6

NA = Not applicable.

## Data Availability

Not applicable.

## Supplementary Materials

The following supporting information can be downloaded at: https://www.mdpi.com/article/10.3390/bios13070748/s1, Figure S1: TEM images of Co-N-C. Figure S2: HAADF-STEM image of Co-N-C and elemental mappings of C, N, Fe, respectively. Figure S3: Raman spectra of carbon black. Figure S4: (a) C 1s and (b) O 1s spectra of Co-N-C. (c), (d) XPS survey spectra of Co-N-C and NC. Figure S5: Experimental verification of the diazotization interaction between oxTMB and NO_2_^−^. Figure S6: Change of the ratiometric colorimetric signal *A*_652_/*A*_445_ along with the diazotization time of oxTMB and NO_2_^−^. Figure S7: (a) UV-vis spectra of the TMB + NO_2_^−^ system with NO_2_^−^ at various levels. (b) Linear relationship between the absorbance at 445 nm and the concentration of NO_2_^−^. Figure S8: Ratiometric colorimetric response of the bimodal ratiometric colorimetric method toward various species. Table S1: Comparison of the TMB kinetic parameters of the Co-N-C with other mimicking enzyme catalysts. Table S2: Comparison of the current work with reported methods for the determination of NO_2_^−^. References [48,49,50,51,52,53,54,55,56,57,58,59,60,61,62,63,64] are cited in the supplementary materials.
