# Supplementary material for: Cobalt–Nitrogen Co-Doped Carbon as Highly Efficient Oxidase Mimics for Colorimetric Assay of Nitrite
Source: Biosensors (Basel). 2023 Jul 20;13(7):748. doi: 10.3390/bios13070748 (PMC10377546; doi:10.3390/bios13070748)
Supplement: Supplementary file 1 [file biosensors-13-00748-s001.zip › biosensors-2431005-supplementary.pdf]

Supporting Information

Cobalt–Nitrogen Co-doped Carbon as highly efficient oxidase mimics for colorimetric assay of nitrite

Dalei Lin [1], Shuzhi Wu [2,*], Shushu Chu [1] and Yizhong Lu [1,*]

[1] *School of Materials Science and Engineering, University of Jinan, Jinan 250022, China*

[2] *Shandong Academy of Preventive Medicine, Shandong Center for Disease Control and Prevention, Jinan, 250014, China*

*Correspondence: wushuzhi@163.com (S.W.); mse_luyz@ujn.edu.cn (Y.L.)

Chemicals

Cobalt (II) acetate tetrahydrate ($(CH_3COO)_2Co\cdot4H_2O$), 1,10-phenanthroline monohydrate, L-histidine, 1,4-dicarboxybenzene (TA), 3,3',5,5'-tetramethylbenzidine (TMB), Superoxide dismutase (SOD), and O-phenylenediamine (OPD) were purchased from Sigma-Aldrich (USA). Ethanol (≥99.7%), Acetic acid (CH_3COOH), and L-Ascorbic acid (AA) were purchased from Sinopharm Chemical Reagent Co., Ltd. 2,2′-azinobis (3-ethylbenzothiazoline-6-sulfonic acid ammonium salt) (ABTS) was purchased from Aladdin Industrial Corp. (Shanghai, China). Potassium thiocyanate (KSCN) was bought from Macklin. All chemicals were used as received without additional purification, and deionized water was used through all the experiments.

Materials characterization

Transmission electron microscope (TEM) images were observed by a JEM-1400 (JEOL Ltd., Japan). Powder X-ray diffraction (XRD) pattern was procured by an Ultima IV diffractometer (Rigku, Japan). The X-ray photoelectron spectroscopy (XPS) was obtained using Thermo Fischer, ESCALAB 250Xi instrument. FTIR spectrometer spectra were recorded using iS50 (Thermofisher Scientific, USA). Brunauer–Emmett–Teller (BET) pore structure and surface area were collected on a Micromeritics 2020 M instrument (Micromeritics, USA). Raman spectra were obtained using Horiba LabRAM HR Evolution (Renishaw, UK). UV-vis spectra analysis and enzyme kinetics tests were collected using a UV-8000 spectrophotometer (Metash, China). Electron spin resonance (ESR) spectra were acquired by a Bruker EMXnano spectrometer (Germany). Fluorescence spectrum analysis was characterized by an RF-6000 Fluorospectrophotometer (Shimadzu, China).

Oxidase-like activity of Co-N-C

The oxidase-like activity of Co-N-C was verified by selecting TMB as a chromogenic substrate. Typically, 20 μL Co-N-C solution (0.5 mg mL^{-1}), 50 μL TMB substrate (5 mM) and 930 μL acetate buffer (0.2 M, pH 3.6) were mixed

comprehensively. Subsequently, after incubation at room temperature for 20 min, the UV-characteristic absorption peak at 652 nm was recorded.

Calculating Kinetics constants

The kinetics constants (K_m and V_{max}) in the Co-N-C + TMB reaction system were calculated by substituting the reaction rate and substrate concentration into the Michaelis–Menten equation as follows:

$$V = \frac{V_{max}[S]}{K_m+[S]} \qquad (1)$$

where V represents the initial chromogenic reaction velocity, V_{max} represents the maximum reaction rate, and K_m represents Michaelis constant, [S] represents the concentration of the substrate (TMB).

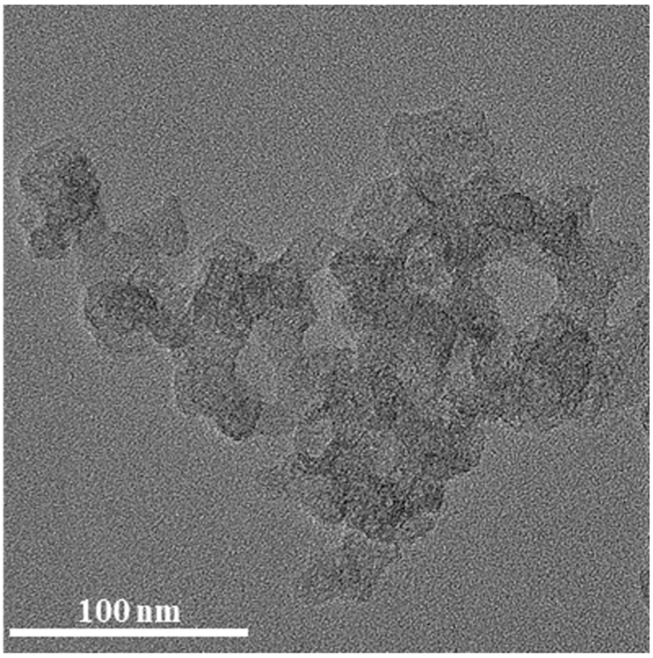

Figure S1. TEM images of Co-N-C.

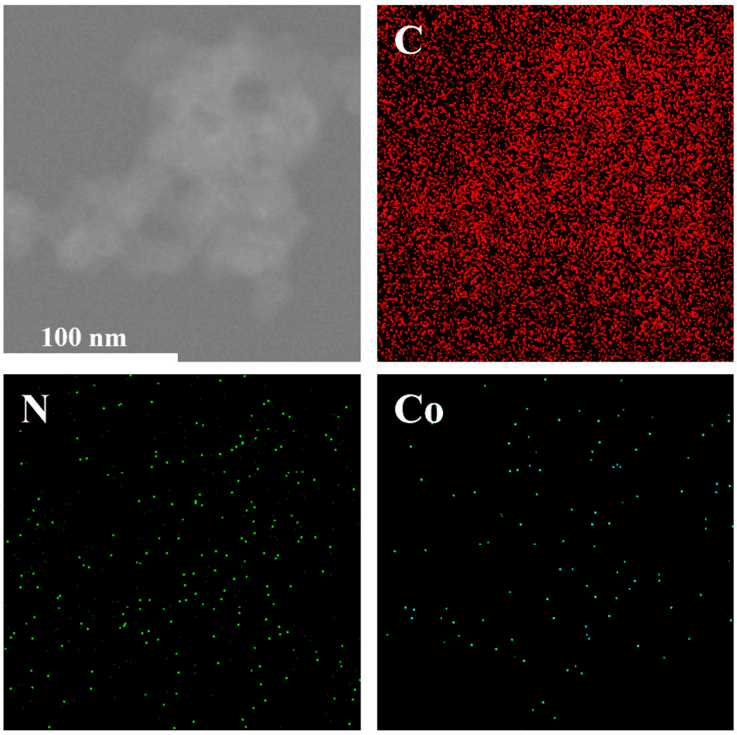

Figure S2. HAADF-STEM image of Co-N-C and elemental mappings of C, N, Fe, respectively.

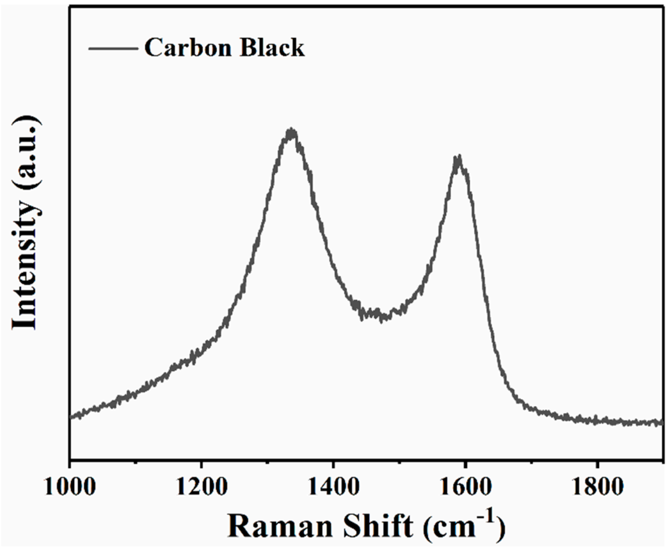

Figure S3. Raman spectra of carbon black.

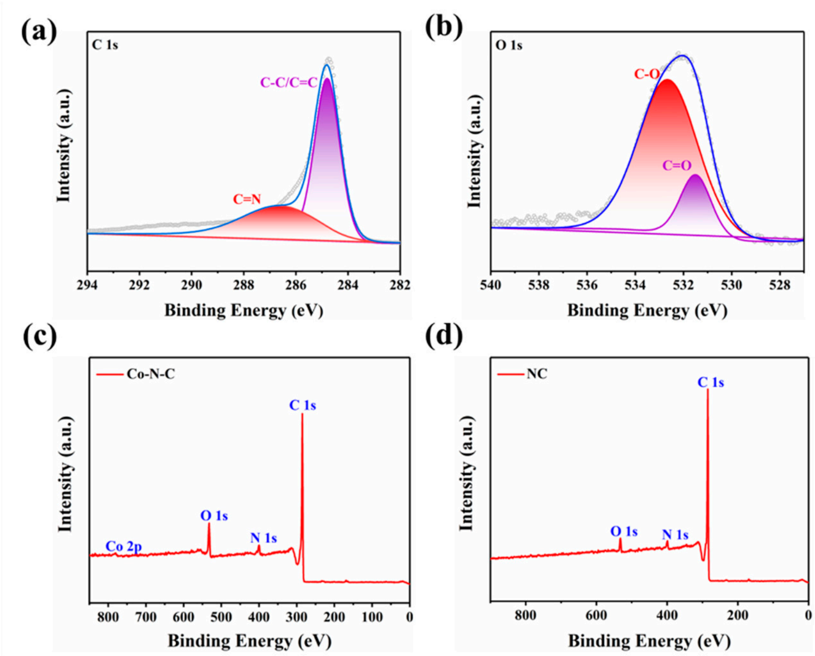

Figure S4. (a) C 1s and (b) O 1s spectra of Co-N-C. (c), (d) XPS survey spectra of Co-N-C and NC.

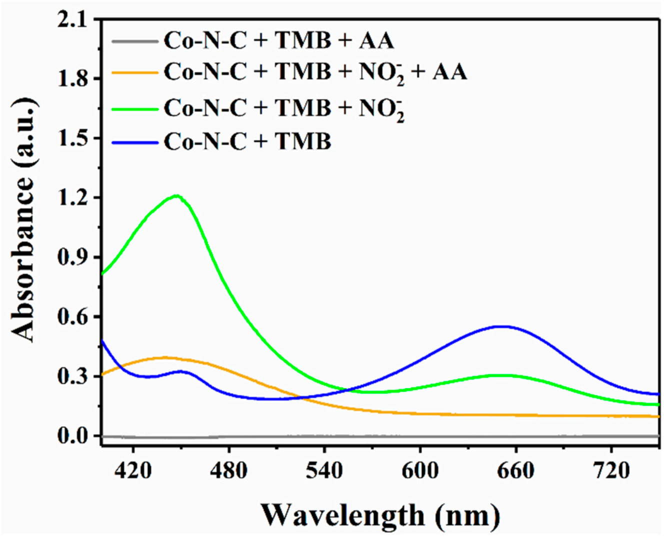

Figure S5. Experimental verification of the diazotization interaction between oxTMB and NO_2^-.

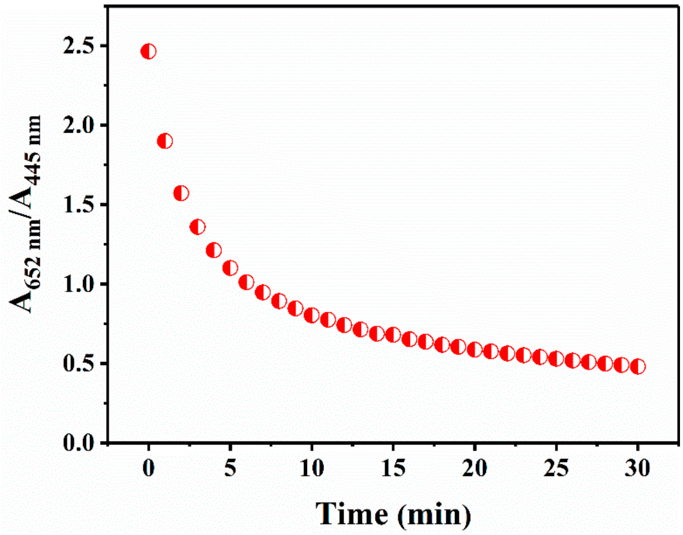

Figure S6. Change of the ratiometric colorimetric signal A_{652}/A_{445} along with the diazotization time of oxTMB and NO_2^-.

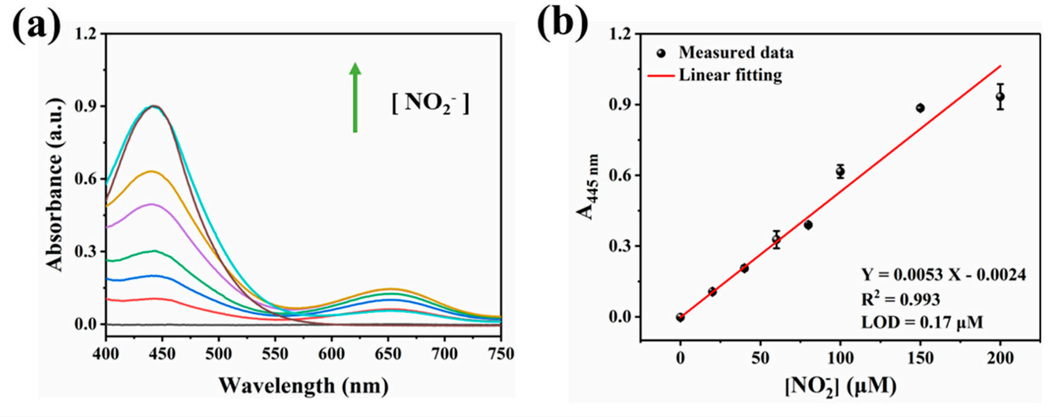

Figure S7. (a) UV-vis spectra of the TMB + NO_2^- system with NO_2^- at various levels. (b) Linear relationship between the absorbance at 445 nm and the concentration of NO_2^-.

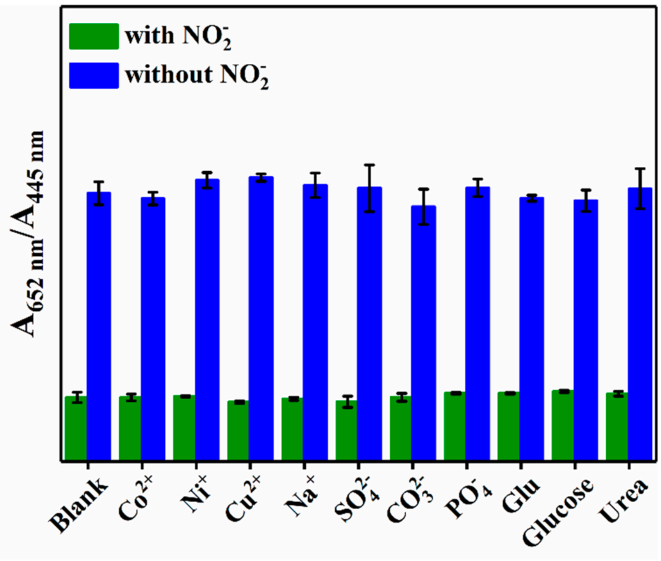

Figure S8. Ratiometric colorimetric response of the bimodal ratiometric colorimetric method toward various species.

Table S1.

Comparison of the TMB kinetic parameters of the Co-N-C with other mimicking enzyme catalysts.

Enzyme	K_m [mM]	v_{max}(10^{-8} M s^{-1})	Ref
Co NPs	1.14	9.98	[1]
$Co_4S_3/Co(OH)_2$	1.33	46.6	[2]
$Fe_{0.5}Co_{0.5}$ nanoparticles	1.79	45.6	[1]
Fe-N-C SAzymes	1.81	0.06	[3]
Co-N-C	0.39	5.79	This work

Table S2

Comparison of the current work with reported methods for the determination of NO_2^-.

Material	Detection method	Detection range (μM)	LOD (μM)	Reference
CTAB-AuNPs	Fluorometric	0.50 - 100	0.17	[4]
MOF@PVP/PVDF	Fluorometric	0.67 - 20	0.67	[5]
Tb-ZrBTB-120	Fluorometric	0.10 - 10	0.08	[6]
ZrT-1-NH_2	Fluorometric	2.50 - 75	0.50	[7]
N-CNDs	Fluorometric	0 - 2000	1	[8]
BSA/MPA-AuNCs	Fluorometric	5 - 30	0.70	[9]
Griess agent	Colorimetric	1.80 - 21.7	0.22	[10]
Hollow MnFeO	Colorimetric	3.30 - 133.3	0.20	[11]
CoOOH	Colorimetric	2.50 - 175	0.09	[12]
Au NSs	Colorimetric	2 - 300	0.40	[13]
Ag-SO_3-NU-902	Electrochemical	0 - 2000	9.10	[14]
SnO_2/Pt/Ti/SiO_2/ Si	Electrochemical	10 - 400	1.70	[15]
CdS/TiO_2	Electrochemical	100 - 500	0.56	[16]
AuNPs/MoS_2/GN	Electrochemical	5 - 5000	1	[17]
Co-N-C	Ratiometric colorimetric	20 - 200	0.039	This work

Reference

[1] CHEN Y, CAO H, SHI W, et al. Fe–Co bimetallic alloy nanoparticles as a highly active peroxidase mimetic and its application in biosensing [J]. *Chemical Communications*, **2013**, 49(44): 5013-5.

[2] WANG J, WANG Y, ZHANG D, et al. Intrinsic oxidase-like nanoenzyme $Co_4S_3/Co(OH)_2$ hybrid nanotubes with broad-spectrum antibacterial activity [J]. *ACS Applied Materials & Interfaces*, **2020**, 12(26): 29614-24.

[3] WU Y, JIAO L, LUO X, et al. Oxidase-like Fe-N-C single-atom nanozymes for the detection of acetylcholinesterase activity [J]. *Small*, **2019**, 15(43): 1903108.

[4] PONLAKHET K, PHOOPLUB K, PHONGSANAM N, et al. Smartphone-based portable fluorescence sensor with gold nanoparticle mediation for selective detection of nitrite ions [J]. *Food Chem*, **2022**, 384: 132478.

[5] ZHANG L, WANG X, KONG W, et al. An enhanced-stability metal–organic framework of NH2-MIL-101 as an improved fluorescent and colorimetric sensor for nitrite detection based on diazotization reaction [J]. *Sensors and Actuators B: Chemical*, **2023**, 386.

[6] CHEN Y-L, SHEN C-H, HUANG C-W, et al. Terbium-modified two-dimensional zirconium-based metal–organic frameworks for photoluminescence detection of nitrite [J]. *Molecular Systems Design & Engineering*, **2023**, 8(3): 330-40.

[7] YANG X, YU X-Y, WANG Q, et al. Metal–organic cages ZrT-1-NH_2 for rapid and selective sensing of nitrite [J]. *Chinese Journal of Analytical Chemistry*, **2023**, 51(2).

[8] ZHANG H, KANG S, WANG G, et al. Fluorescence determination of nitrite in water using prawn-shell derived nitrogen-doped carbon nanodots as fluorophores [J]. *ACS Sensors*, **2016**, 1(7): 875-81.

[9] DENG H-H, HUANG K-Y, ZHANG M-J, et al. Sensitive and selective nitrite assay based on fluorescent gold nanoclusters and Fe^{2+}/Fe^{3+} redox reaction [J]. *Food Chemistry*, **2020**, 317: 126456.

[10] POL R, DIEZ L, GABRIEL D, et al. Versatile three-dimensional-printed platform for nitrite ion analyses using a smartphone with real-time location [J]. *Analytical Chemistry*, **2019**, 91(21): 13916-23.

[11] WANG M, LIU P, ZHU H, et al. Ratiometric colorimetric detection of nitrite realized by stringing nanozyme catalysis and diazotization together [J]. *Biosensors (Basel)*, **2021**, 11(8).

[12] ZHANG J, CHEN H, LIU J, et al. The target-induced redox and diazotized reaction for colorimetric ratio detection of nitrite using CoOOH nanosheets as mimetic oxidase [J]. *Talanta*, **2023**, 258: 124458.

[13] HONG C, LI D, CAO S, et al. Sensitive and multicolor detection of nitrite based on iodide-mediated etching of gold nanostars [J]. *Chemical Communications*, **2022**, 58(93): 12983-6.

[14] WANG Y-C, CHEN Y-C, CHUANG W-S, et al. Pore-confined silver nanoparticles in a porphyrinic metal–organic framework for electrochemical nitrite detection [J]. *ACS Applied Nano Materials*, **2020**, 3(9): 9440-8.

[15] LETE C, CHELU M, MARIN M, et al. Nitrite electrochemical sensing platform based on tin oxide films [J]. *Sensors and Actuators B: Chemical*, **2020**, 316.

[16] GAO B, ZHAO X, LIANG Z, et al. CdS/TiO_2 nanocomposite-based photoelectrochemical sensor for a sensitive determination of nitrite in principle of etching reaction [J]. *Analytical Chemistry*, **2021**, 93(2): 820-7.

[17] HAN Y, ZHANG R, DONG C, et al. Sensitive electrochemical sensor for nitrite ions based on

rose-like AuNPs/MoS_2/graphene composite [J]. *Biosensors and Bioelectronics*, **2019**, 142: 111529.
